# Reduced motivation to work for financial reward associated with harmful alcohol use in a community sample of young adults

**DOI:** 10.1192/bjo.2021.683

**Published:** 2021-06-18

**Authors:** Jessica Henry, Karen Ersche, Tsen Vei Lim

**Affiliations:** Department of Psychiatry, University of Cambridge

## Abstract

**Aims:**

Neuroimaging research suggests that alcohol dependency is associated with impairments in anticipating monetary rewards, but not aversive or alcohol-related cues.

We sought to investigate if reinforcement sensitivity is altered in young adults, who regularly consume harmful levels of alcohol, using a monetary incentive reinforcement (MIR) task. In light of previous research suggesting reduced motivation to obtain reward, we hypothesized that young alcohol users would show reduced motivation for monetary gain, but unimpaired loss avoidance behaviour.

**Method:**

We recruited 46 volunteers from the local community in Cambridge (UK), half of whom reported consuming alcohol at harmful levels, as reflected by the Alcohol Use Disorder Test. Participants completed a number of personality questionnaires, including the Barratt Impulsivity Scale (BIS-11) and Sensation-Seeking-Scale (SSS-V) and performed the MIR task, which measures participants’ efforts in avoiding punishment and gaining rewards. Data were analysed using Statistical Package for Social Sciences (SPSS) version 25 (IBM, Chicago IL). Analysis of co-variance (ANOVA) were used to explore group differences in demographics, personality traits and task performance; age and gender were included as co-variates.

**Result:**

The groups were well-matched in terms of socioeconomic status and education levels. As the alcohol group was significantly younger than the control group and dominated by females, age and gender were statistically controlled for. Alcohol users reported significantly higher levels of impulsivity (F1,41 = 6.0, p = 0.019) and sensation-seeking traits (F1,42 = 36.7, p < 0.001) and demonstrated normal sensitivity to monetary value (F1,41 = 1.07,p = 0.307). However, when challenged to on the MIR task to gain reward or avoid punishment, alcohol users were as equally motivated as control volunteers to take action to avoid financial loss (F1,41 = 2.6,p = 0.112) but showed less motivation to work towards financial reward (F1,41 = 4.7,p = 0.036). Especially for small rewards, alcohol users exerted significantly less efforts, as reflected by a reduced accuracy rate (F1,41 = 6.6,p = 0.014) and a significant increase in late responses (F1,41 = 7.7,p = 0.008). The lack of motivation to work for reward was negatively associated with the severity of alcohol use, as reflected by the AUDIT score (r=−.48,p < 0-05).

**Conclusion:**

We observed reduced motivation to obtain financial reward, but not avoid loss in a community sample of heavy drinkers. As the observed effect was directly related to alcohol use severity, it may suggest changes in reinforcement sensitivity occur at an early stage of chronic alcohol use. Future research may want to monitor reward motivation in alcohol users longitudinally to evaluate whether it would be a suitable target for early intervention.

